# A Cell Metamodel Uncovers Mechanistic Drivers of Disease Phenotypes Across Molecular, Cellular, and Tissue Scales

**DOI:** 10.21203/rs.3.rs-7731109/v1

**Published:** 2025-11-28

**Authors:** Chenxi Wang, Jingjing Zheng, Weimin Li, Xianni Zhong, Bing Yu, Angdi Li, Junjie He, Xiaoyan Liu, Qifeng Liao, Andrej Sali, Xuming He, Liping Sun

**Affiliations:** 1 iHuman Institute, ShanghaiTech University, Shanghai 201210, China; 2 School of Life Science and Technology, ShanghaiTech University, Shanghai 201210, China; 3 National Key Laboratory of Biomacromolecules, CAS Center for Excellence in Biomacromolecules, Institute of Biophysics, Chinese Academy of Sciences, Beijing, China; 4 School of Information Science and Technology, ShanghaiTech University, Shanghai 201210, China; 5 Department of Bioengineering and Therapeutic Sciences, University of California, San Francisco, CA 94158; 6 Department of Pharmaceutical Sciences, University of California, San Francisco, CA 94158; 7 Quantitative Biosciences Institute, University of California, San Francisco, CA 94158; 8 Shanghai Engineering Research Center of Intelligent Vision and Imaging, Shanghai 201210, China

## Abstract

Understanding how molecular and cellular dynamics regulate tissue function remains a central challenge in biology. Here, we develop a graph-based metamodeling framework for modeling of complex biological systems and apply it to construct a comprehensive metamodel of the β-cell. The metamodel integrates 115 input models that describe various aspects of β-cell physiology across molecular, cellular, and multicellular spatial scales, spanning ten orders of magnitude in timescales. Validated against a broad range of experimental data, including fluorescence imaging, the metamodel uncovers how the interplay between gap junction coupling and $K^{+}$ channel–mediated signaling regulates islet function. Moreover, the metamodel identifies and characterizes two types of hub cells, each deterministically driving islet activity and synchronization *via* unique ion channel properties. Perturbing channel conductance and hub cell activity recapitulates distinct diabetic phenotypes, highlighting them as mechanistic drivers of diabetes and potential therapeutic targets. Our metamodeling framework is broadly applicable to modeling other complex biological systems.

## Introduction

Cells are involved in biological processes across multiple scales, including molecular processes occurring at the atomic level over milliseconds, cellular dynamics unfolding over seconds, and multicellular interactions spanning minutes. This multiscale complexity underlies the remarkable adaptability of cells to diverse perturbations. It also poses a major challenge to decipher how perturbations at one scale propagate to other scales and ultimately drive cell function. This challenge can often be addressed with the aid of computational models that can uncover the regulatory mechanisms governing cellular behavior in both health and disease. Such models are generally built based on information from experiments, physical theories, statistical inferences, and/or other prior models [1, 2]. The most accurate, precise, and complete models are computed by integrating all available information.

Correspondingly, the integrative approach has significantly advanced the development of cell models. Some notable efforts include CellPACK [3, 4], which integrates structural and systems biology data to model mesoscale cell architecture; Lattice Microbes [5], which couples spatially resolved simulations with chemical reaction networks to explore RNA splicing; Vivarium [6, 7], which combines submodels of cellular function *via* ordinary differential equations (ODEs) to predict cellular behaviors; DCell [8, 9], which integrates prior knowledge and data using deep neural networks to construct hierarchical cell structures; and Bayesian metamodeling [1], developed in our previous work, which simplifies and integrates a collection of models using dynamic Bayesian networks to reveal multiscale cell dynamics. However, despite these advances, current approaches often (i) oversimplify inputs through coarse-graining and linearization, reducing model accuracy; (ii) neglect uncertainty, particularly in ODE-based models, undermining estimates of model precision and reliability; (iii) rely on *ad hoc* coupling strategies limited to common timesteps using, for example, least-squares methods, introducing bias and reducing coupling efficiency; and (iv) use inefficient inference algorithms for model interpretation, reducing the scalability to highly complex systems. Overcoming these limitations is essential for building comprehensive and interpretable cell models capable of formulating testable biological predictions.

To address these limitations, we develop a graph-based metamodeling framework that integrates models across multiple representations, spatial domains, and time scales. Leveraging probabilistic graphical models, graph-based metamodeling accurately converts diverse input models into a universal representation, while preserving their high-order nonlinear complexity, incorporating experimental data, and rigorously propagating and estimating uncertainty. To effectively capture interactions across time scales, graph-based metamodeling employs a combination of nonlinear and linear mappings to unify temporal resolutions, supplemented by a standardized coupling strategy to mitigate biases introduced by *ad hoc* coupling. Moreover, it enables efficient model inference using factor graphs to facilitate message passing across complex model structures and approximation algorithms tailored to specific variable distributions. The resulting metamodel attains higher accuracy, precision, and completeness than the input models, while maintaining computational efficiency and scalability, thereby providing deeper insights than those achievable with the input models alone.

Here, we apply graph-based metamodeling to pancreatic β-cells, integrating 57 vesicle exocytosis models, 57 insulin secretion kinetics models, and a network model of intercellular communication within the islet. The resulting metamodel includes approximately 1,500 variables and 3,300 parameters, capturing glucose-stimulated insulin secretion at the islet level, while also resolving the molecular and cellular dynamics of individual β-cells. Following extensive validation against experimental data, including in-house fluorescence imaging of cellular structures, we uncover an interplay between gap junction–coordinated islet synchronization and $K^{+}$ channel–mediated single-cell dynamics that regulates biphasic, oscillatory insulin secretion. Perturbations of channel conductance recapitulate distinct diabetic phenotypes, indicating that modulating the balance between gap junction coupling and $K^{+}$ channel-mediated signaling might hold the key to restoring normal insulin secretion. Moreover, we identify and characterize two types of hub cells with distinct ion channel properties, each deterministically driving islet activity and synchronization. Silencing them reproduces hallmark features of type 2 diabetes, suggesting that promoting hub cell emergence could offer a potential therapeutic strategy to recover islet function. By linking molecular and cellular perturbations with islet dysfunction in diabetic phenotypes, metamodeling uncovers mechanistic drivers of diabetes and highlights potential therapeutic targets.

## Results

### A graph-based metamodel bridging molecular and cellular dynamics to emergent islet function

**Fig 1.**
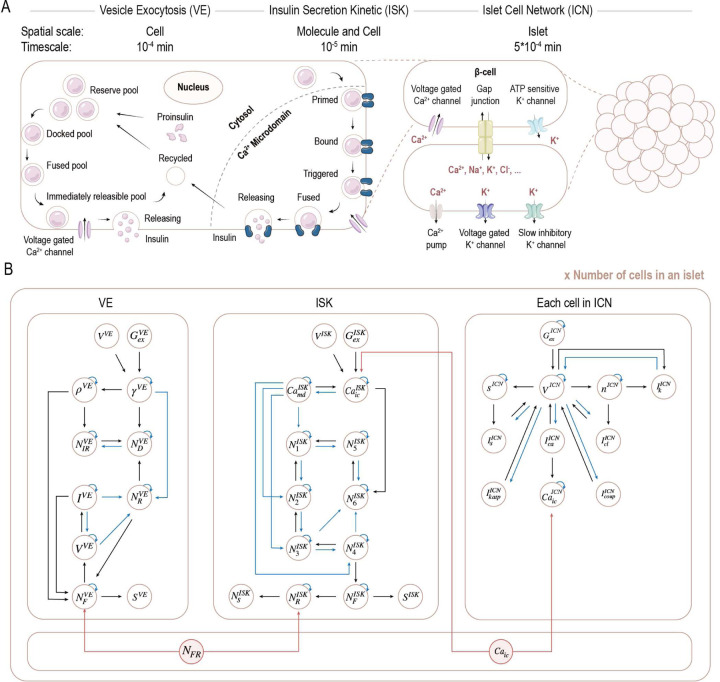
Graph-based multiscale β-cell metamodel (A) Three input models describing distinct aspects of the β-cell across varying spatial and temporal scales: the Vesicle Exocytosis (VE) model, the Insulin Secretion Kinetic (ISK) model, and the Islet Cell Network (ICN) model. (B) Meta PGM graph for each cell in the metamodel. Variables are represented as white circles outlined in light brown. Directed edges, shown as black and blue arrows, indicate intra- and inter-timestep statistical dependencies between parent and child variables, respectively. Self-loops in blue indicate inter-timestep dependence on the same variable from the previous timestep. Coupling variables are represented as nodes in light pink circles outlined in red, with red edges denoting dependencies introduced through coupling.

Cell systems are often described by various models, each capturing distinct aspects of the system. These models typically feature high-dimensional, nonlinear dynamics with interdependent variables spanning diverse representations and spatiotemporal scales. To gain quantitative insights into the mechanisms underlying pancreatic β-cell dynamics and islet function, we construct a cell metamodel by integrating three types of models (Fig. 1A, Methods): (i) Vesicle Exocytosis (VE) model (Spatial scale: Cell; Timescale: 10^−4^ min), ODE-based model describing insulin vesicle trafficking across various pools and their exocytosis in single β-cells [10] (SI Text 1.1, SI Table S1); (ii) Insulin Secretion Kinetic (ISK) model (Spatial scale: Molecule and Cell; Timescale: 10^−5^ min), compartment-based model capturing $Ca^{2+}$-dependent insulin secretion kinetics in single β-cells [11] (SI Text 1.2, SI Table S2); and (iii) Islet Cell Network (ICN) model (Spatial scale: Islet; Timescale: 5 × 10^−4^ min), network-based model describing electrical activity, ${Ca}^{2+}$ dynamics, and intercellular communications within an islet [12] (SI Text 1.3, SI Table S3). For models lacking uncertainty quantification, we estimate probability distributions from the experimental data used to construct them (SI Text 2). We then standardize physiological conditions across input models and integrate them into a multiscale cell metamodel using our graph-based metamodeling framework (SI Fig. S1, Methods).

First, we convert each input model into a universal probabilistic surrogate, represented as a Probabilistic Graphical Model (PGM) (Fig. 1B; SI Text 1.1–1.3). The surrogate model describes intra- and inter-timestep statistical dependencies (represented as edges in black and blue, respectively) between state variables (represented as nodes outlined in light brown). Specifically, we apply State-Space Model (SSM) to capture complex dynamics through state transitions using (i) a process model, which employs input model ODEs as forward functions, and is corrupted by transition noise to account for intrinsic stochasticity; and (ii) an observation model, which uses experimental data when available or an identical function to align predicted and observed states, and is also corrupted by emission noise to capture observation uncertainty. This conversion preserves high-order nonlinear dependencies among model variables, incorporates experimental data to refine variable estimates, and rigorously propagates uncertainty, ensuring reliable surrogate representations of diverse input models.

Next, we use nonlinear and linear mappings to align all models to a universal timestep of 10^−4^ min. The original timesteps of the input models were VE: 10^−4^ min, ISK: 10^−4^ min, and ICN: 5 × 10^−4^ min. The VE surrogate model requires no redefinition, as its timestep already matches the universal timestep. For the ISK surrogate model, the process model is redefined at the universal timestep using the forward function, while the observation model is directly derived from the original observations at smaller timesteps. Similarly, the ICN process model is adjusted to the universal timestep, while the observation model was redefined by linearly interpolating experimental data. The coupling PGM graph is then constructed for the process model at each universal timestep using a standardized strategy (Fig. 1B). We identify two sets of connecting variables from different surrogate models: (i) the number of insulin vesicles fused with membrane in VE model, $N_{F}^{V\;E}$, and those in the releasable state in ISK model, $N_{R}^{ISK}$, which exhibit the highest Pearson correlation coefficient among all pairs of surrogate model variables [13]; (ii) the intracellular $Ca^{2+}$ levels in ISK, $Ca_{ic}^{ISK}$, and ICN models, $Ca_{ic}^{ICN}$, which share identical semantics (SI Fig. S2). We introduce two explicit coupling variables, $N^{F\;R}$ and $Ca_{ic}$, whose prior distributions are defined as mixtures of the corresponding connecting variable distributions, with equal weights assigned in the absence of prior knowledge about relative model confidence. Finally, to establish the statistical dependencies, we construct coupling PGM graphs by introducing four directed edges from the coupling variables to their respective connected variables: $N_{F}^{V\;E}\leftarrow N_{F\;R}\to N_{R}^{ISK}$ and $Ca_{ic}^{ISK}\leftarrow Ca_{ic}\to Ca_{ic}^{ICN}$. The conditional probability distributions are parameterized based on the relative overlaps between the coupling and connecting variable distributions: $P\left(N_{FR}|N_{F}^{V\;E}\right),P\left(N_{F\;R}|N_{R}^{ISK}\right),P\left(Ca_{ic}|Ca_{ic}^{ISK}\right)$, and $P\left(Ca_{ic}|Ca_{ic}^{ICN}\right)$. Thus, we obtain a meta PGM graph that includes all surrogate model variables and coupling variables. This coupling strategy unifies temporal resolution through linear and nonlinear mappings, rather than relying solely on shared timesteps, and applies a standardized approach to mitigate biases from *ad hoc* coupling, accurately capturing cross-timescale interactions among surrogates.

Last, we perform approximate inference over the metamodel using factor graphs, allowing message passing through the complex structures of the metamodel. We introduce factors to capture subsets of distributions and statistical dependencies between state and coupling variables, with the metamodel distribution calculated as the product of all these factors. The state variable distributions are then estimated by recursively applying the *predict–update* cycle within and across timesteps using either Unscented Kalman Filters (UKFs). This inference approach leverages factor graphs and approximate techniques tailored to specific variable distributions, enabling efficient interpretation of the metamodel. The graph-based metamodeling framework has been rigorously validated with a synthetic metamodel of glucose-stimulated insulin secretion (SI Text 3, SI Fig. S3), demonstrating its ability to integrate diverse data and models to study complex dynamic systems.

### The cell metamodel recapitalizes glucose-stimulated insulin secretion across scales

Glucose-stimulated insulin secretion in pancreatic β-cells is a multiscale process involving molecular pathways, cellular dynamics, and intercellular interactions. At the molecular scale, glucose stimulation increases the ATP-to-ADP ratio, triggering the closure of ATP-sensitive $K^{+}\left({\text{K}}_{ATP}\right)$ channels, leading to membrane depolarization, ${Ca}^{2+}$ influx and subsequent insulin secretion [14]. At the cellular scale, insulin vesicles are populated into distinct pools: the readily releasable pool and the reserve pool, driving the rapid first-phase and the prolonged second-phase of insulin secretion, respectively [15, 16]. Within RRP, a subset of vesicles, the immediately releasable pool (IRP), is primed and docked at the plasma membrane, ready for immediate exocytosis upon glucose stimulation. At the islet level, ion exchanges between β-cells regulate their dynamics and synchronization, driving biphasic, oscillatory insulin secretion that is essential for hepatic insulin sensitivity and glucose homeostasis [17, 18]. In type 2 diabetes (T2D), insulin secretion is impaired, marked by a reduced first-phase response and disrupted oscillatory patterns, typically with higher frequency and lower amplitude pulses [19, 20]. Restoring the physiological pattern of islet insulin secretion is, therefore, a central therapeutic goal in T2D treatments.

**Fig 2.**
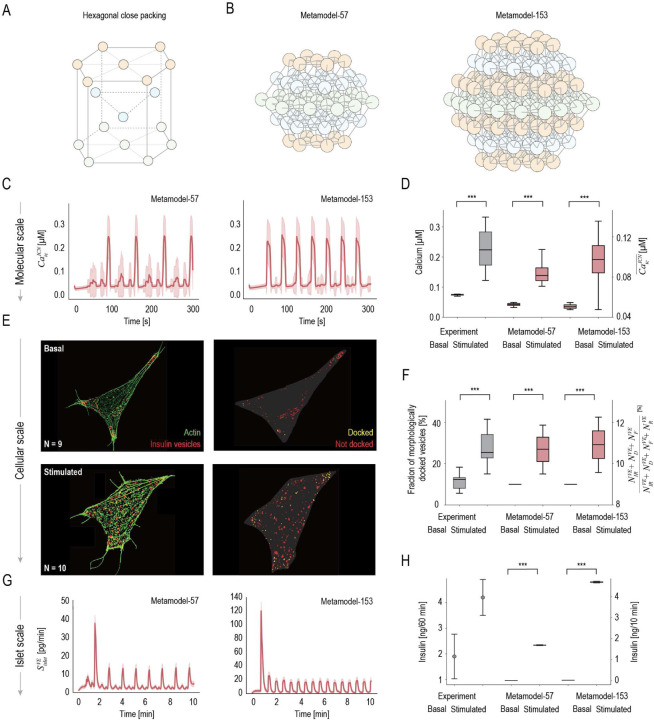
Multiscale β-cell metamodel under basal and glucose-stimulated conditions (A) Hexagonal close packing of β-cells (white circles outlined in black) in the islet network. (B) Side views of islet networks of Metamodel-57 and Metamodel-153, comprising 57 and 153 β-cells (white circles outlined in black), respectively. β-cells in different layers are shown in different colors, with adjacent cells connected by gray solid lines. (C) Intracellular ${Ca}^{2+}$ levels of all cells in Metamodel-57 and Metamodel-153 over 300 seconds upon glucose stimulation. (D)Intracellular $Ca^{2+}$ levels measured under basal (G5) and glucose-stimulated (G10) conditions [21] are compared with predictions from two metamodels, with the predictions averaged over 10 minutes for both conditions. (E) Structured Illumination Microscopy (SIM) images showing actin filaments (green) and insulin vesicles (red) in INS-1E β-cells under basal and glucose-stimulated conditions. Morphologically docked insulin vesicles are indicated in yellow, while undocked ones are shown in red. (F) The fraction of morphologically docked insulin vesicles measured by SIM (gray), compared with the fraction of insulin vesicles in the immediately releasable pool (IRP), $N_{IR}^{V\;E}$, docked pool, $N_{D}^{V\;E}$, and fused pool, $N_{F}^{V\;E}$ relative to the total vesicle population (including the reserve pool, $N_{R}^{V\;E}$) predicted by both metamodels (red) under basal and glucose-stimulated conditions. (G) Islet insulin secretion rate, $S_{\text{islet}}^{VE}$, predicted by both metamodels. (D) Experimental measurements of insulin secretion from an islet over 60 minutes (gray) [22], compared with metamodel predictions from one islet over 10 minutes (red) under basal and glucose-stimulated conditions. Experimental data are shown as gray dots, with error bars representing standard deviations (SDs), as raw data were unavailable and were not subjected to t-test analysis. Shaded regions represent SDs. Statistical significance was assessed using t-tests and is indicated as follows: * p < 0.05, **p < 0.01, ***p < 0.001.

We construct two β-cell metamodels and examine their ability to capture molecular dynamics, cellular structure, and islet insulin secretion under basal and glucose-stimulated conditions. While typical human islets contains approximately 500 to 2,400 β-cells [23], smaller islet sizes are used for computational efficiency. The islet network is modeled as a hexagonal close-packed lattice (Fig. 2A), with a central layer of 3 or 5 cells per edge, flanked by alternating regular and irregular layers, yielding networks of 57 or 153 β-cells. Metamodel-57 represents an islet composed of 57 β-cells, incorporating 1,482 surrogate model variables, 114 coupling variables, and 3,306 parameters (Fig. 2B). Metamodel-153 represents a larger islet with 153 β-cells, comprising 4,743 surrogate model variables, 306 coupling variables, and 13,005 parameters (Fig. 2C). Both metamodels, constructed in a healthy islet environment, uses conductance values of approximately 5–15 pS for GJs, 145 pS for ${\text{K}}_{ATP}$ channels, and 1000 pS for ${Ca}^{2+}$ channels [24, 25]. To reflect β-cell heterogeneity within the islet network, individual cell conductance values are sampled from a normal distribution with a 5–10% standard deviation (SI Fig. S4). At the molecular scale (Fig. 2C), both metamodels replicate intracellular ${Ca}^{2+}$ oscillations in response to glucose stimulation, exhibiting amplitudes (0-0.3μM) and periods (15–150 s) that closely match microspectrofluorometry experiments [21]. The observed increases in average $Ca^{2+}$ levels under basal and glucose-stimulated conditions also align well with experimental data (Fig. 2D) [21]. At the cellular scale, we conducted Structured Illumination Microscopy (SIM) imaging of INS-1E β-cells to segment and classify morphologically docked insulin vesicles under basal and glucose-stimulated conditions (Fig. 2E, Methods). The metamodel predicts increased fraction of insulin vesicles in the IRP, $N_{IR}^{V\;E}$, docked pool, $N_{D}^{V\;E}$, and fused pool, $N_{F}^{V\;E}$, consistent with SIM observations showing a higher fraction of morphologically docked vesicles upon glucose stimulation (Fig. 2F). At the islet scale, both metamodels predict a biphasic, oscillatory insulin secretion in response to glucose (Fig. 2G). Metamodel-153 produces a more pronounced initial peak followed by more frequent but lower-amplitude oscillations compared to Metamodel-57. Note that the first-phase insulin secretion in both metamodels ends within the first 3 minutes, which is shorter than the 10-minute duration typically observed experimentally [26]. This difference might arise from the simplified islet structure, limited cell number, and the exclusion of other endocrine cells (*eg*, α-cells) in the current metamodel. Nonetheless, the increase in the total insulin secretion under basal and glucose-stimulated conditions predicted by the metamodels agrees well with measurements using mouse islet [22] (Fig. 2H). Together, these results validate the cell metamodel across scales, with Metamodel-57 used in subsequent analyses for computational efficiency.

### Balancing gap junction coupling and $K^{+}$ channel–mediated signaling to regulate islet function

**Fig 3.**
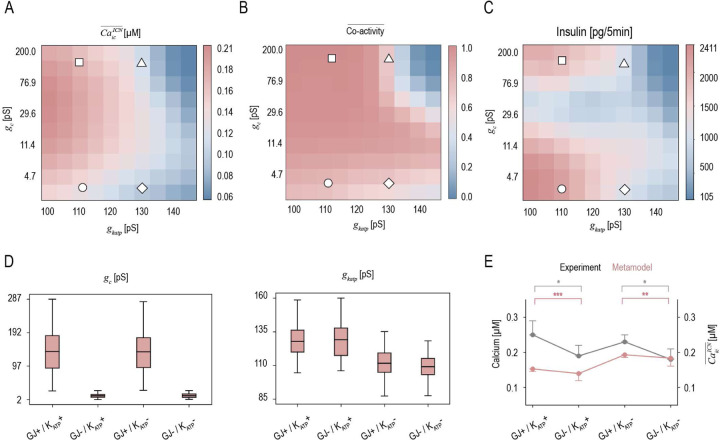
Multiscale β-cell metamodel under varying intercellular communication conditions (A-C) Changes in average intracellular $Ca^{2+}$ level, $\bar{Ca_{ic}^{ICN}}$, cell co-activity and insulin secretion from the islet over 300 seconds of glucose stimulation under varying gap junctions (GJ) conductance ($g_{c}$, ranging from 4 to 200 pS), and ${\text{K}}_{ATP}$ channel conductance ($g_{\text{katp}}$, ranging from 100 to 145 pS). (D) Parameters used to define four combinations of elevated (+) and lowered (−) GJ and ${\text{K}}_{ATP}$ channel conductance in the metamodel: $\text{GJ}+/{\text{K}}_{ATP}+$, $\text{GJ}-/{\text{K}}_{ATP}+$, $\text{GJ}+/{\text{K}}_{ATP^{-}}$ and $\text{GJ}-/{\text{K}}_{ATP^{-}}$. For $\text{GJ}+/{\text{K}}_{ATP}+,g_{c}$ is sampled from N(155,15) and $g_{\text{katp}}$ from N(130,13). For $\text{GJ}-/{\text{K}}_{ATP}+,g_{c}$ is sampled from N(15,1.5) and $g_{\text{katp}}$ from N(130,13). For $\text{GJ}+/{\text{K}}_{ATP^{-}},g_{c}$ is sampled from N(155,15) and $g_{\text{katp}}$ from N(110,11). For $\text{GJ}-/{\text{K}}_{ATP^{-}},g_{c}$ is sampled from N(15,1.5) and $g_{\text{katp}}$ from N(110,11). (E) Experimental measurements of intracellular ${Ca}^{2+}$ levels [22] (10 minutes after glucose stimulation, gray) compared to predictions from the metamodel (5 minutes after glucose stimulation, red) under the four intercellular communication conditions. Error bars represent standard deviations (SDs). Statistical significance was assessed using t-tests and is indicated as follows: *p < 0.05, **p < 0.01, ***p < 0.001.

Intercellular communication, facilitated by electrical coupling through gap junctions (GJs) and metabolic electrical signaling *via* $K^{+}$ channels, plays a critical role in regulating β-cell dynamics and islet function [27, 22]. Using the metamodel, we examine how these mechanisms propagate across scales to regulate islet insulin secretion under glucose stimulation, considering extreme variations in gap junction conductance, $g_{c}$, and ${\text{K}}_{ATP}$ channel conductance, $g_{\text{katp}}$ [28, 29]. A cell is considered active when its peak $Ca^{2+}$ level exceeds 0.15μM, and co-activity between cell pairs is quantified as the duration of their simultaneous activity, normalized by the square root of the product of their individual active durations. The average intracellular $Ca^{2+}$ level, $\bar{Ca_{ic}^{ICN}}$, strongly negatively correlates with $g_{\text{katp}}$ (Fig. 3A). β-cells exhibit high co-activity except when both gap junction and $K_{\text{ATP}}$ channel conductances are elevated, in which case only a few cells remain active (Fig. 3B). Total islet insulin secretion is modulated by the interplay between $g_{c}$ and $g_{\text{katp}}$ (Fig. 3C): when $g_{c}$ exceeds 20 pS, secretion increases with $g_{c}$ and decreases with $g_{\text{katp}}$; below 20 pS, secretion shows a negative correlation with $g_{\text{katp}}$. We then perturb GJ and ${\text{K}}_{ATP}$ channel conductances to create four conditions, excluding extreme values to avoid total islet dysfunction: $\text{GJ}+/{\text{K}}_{ATP}+,\text{GJ}-/{\text{K}}_{ATP}+,\text{GJ}+/{\text{K}}_{ATP^{-}},\text{GJ}-/{\text{K}}_{ATP^{-}}$ (Fig. 3D). Individual β-cell channel conductances are sampled from Gaussian distributions with a 10% standard deviation. Mean GJ+/− values differ by 10-fold, consistent with single-cell clamp recordings [22], while mean ${\text{K}}_{ATP}+/-$ values reflect conductance increases observed in Kir6.2 mutants, the pore-forming subunit of ${\text{K}}_{ATP}$ channels, relative to healthy β-cells [28]. These conductances are validated by reproducing the observed drop in intracellular $Ca^{2+}$ levels when comparing $\text{GJ}+/{\text{K}}_{ATP}+$ with $\text{GJ}-/{\text{K}}_{ATP}+$, and $\text{GJ}+/{\text{K}}_{ATP^{-}}$ with $\text{GJ}-/{\text{K}}_{ATP^{-}}$, as measured by fluorescence microscopy [22] (Fig. 3E).

**Fig 4.**
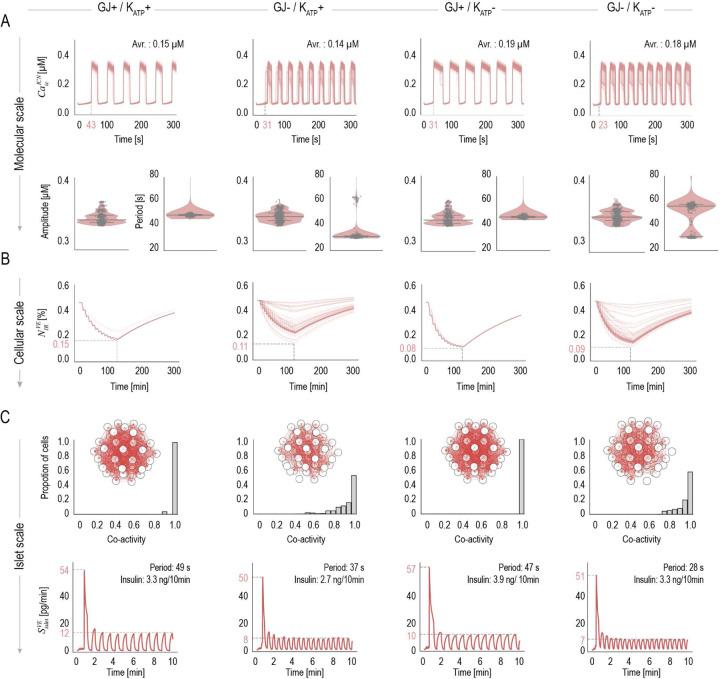
Effects of varying gap junction coupling and $K_{ATP}$-mediated signaling across scales Metamodel predictions across molecular, cellular and islet scales under four intercellular communication conditions, including (A) Intracellular $Ca^{2+}$ level of individual cells, $Ca_{ic}^{ICN}$, (B) The fraction of immediately releasable insulin vesicles, $N_{IR}^{VE}$, in individual cells, and (C) Proportion of cells exhibiting varying levels of co-activity with others, identification of cell pairs with co-activity >0.95 within the islet, and the insulin secretion rate of a single islet, $S_{\text{islet}}^{V\;E}$. Error bars and shaded regions represent standard deviations (SDs). Statistical significance was assessed using t-tests and is indicated as follows: *p < 0.05, **p < 0.01, ***p < 0.001.

We further compare changes in intracellular $Ca^{2+}$ levels, $Ca_{ic}^{ICN}$, the fraction of IRP vesicles, $N_{IR}^{V\;E}$, the degree of islet synchronization *via* cell co-activity, islet insulin secretion rate, $S_{\text{islet}}^{V\;E}$, and other metamodel variables across varying channel conductances (Fig. 4, SI Fig. S5). Under $\text{GJ}+/{\text{K}}_{ATP}+$ conditions, ${Ca}^{2+}$ oscillations are regular, IRP vesicles initially decrease and then gradually increase, cell pairs display high co-activity (> 0.85), and insulin secretion exhibits a biphasic, oscillatory pattern. Perturbing GJ conductance $\left(\text{GJ}-/{\text{K}}_{ATP}+\right)$ accelerates and destabilizes $Ca^{2+}$ oscillations (Fig. 4A), which in turn disrupt IRP vesicle dynamics (Fig. 4B) and reduce islet synchronization, with more β-cells shifting to lower levels of co-activity (Fig. 4C). Insulin secretion exhibits a decreased first-phase peak, a disrupted second-phase oscillatory pattern characterized by lower amplitudes and shorter periods, and an overall reduction in secretion amount (Fig. 4C). These results demonstrate that GJs mechanistically link β-cells to coordinate islet synchronization and function. In contrast, reducing ${\text{K}}_{ATP}$ conductance $\left(\text{GJ}+/{\text{K}}_{ATP^{-}}\right)$ reveals an alternative regulatory mechanism. ${\text{K}}_{ATP}$ inhibition enhances cell excitability by accelerating the onset and prolonging the duration of each depolarized phase (Fig. 4A). This temporal modulation likely sustains β-cell responses to glucose while maintaining ${Ca}^{2+}$ homeostasis. It is accompanied by ultra-rapid and coordinated IRP vesicle release and replenishment, suggesting enhanced insulin vesicle turnover (Fig. 4B). Notably, islet synchronization is markedly enhanced, with all cell pairs exhibiting high co-activity (> 0.95) (Fig. 4C). The first-phase insulin secretion peak is amplified, subsequent peaks become broader in duration but slightly reduced in amplitude, leading to an overall increase in total insulin secretion. These findings indicate that ${\text{K}}_{ATP}$ channels temporally modulate the oscillatory dynamics of β cells to regulate islet insulin secretion.

When both GJ and ${\text{K}}_{ATP}$ channel conductance are reduced $\left(\text{GJ}-/{\text{K}}_{ATP^{-}}\right)$, the metamodel reveals an emergent interplay between these two opposing regulatory mechanisms. Although ${Ca}^{2+}$ oscillations become irregular due to weak intercellular coupling, the duration of each depolarized phase is prolonged, leading to an overall elevated intracellular ${Ca}^{2+}$ levels (Fig. 4A). Insulin vesicle release remains rapid but still lacks coordination (Fig. 4B), and overall islet synchronization is diminished, though partially rescued compared to $\text{GJ}-/{\text{K}}_{ATP}+$ conditions (Fig. 4C). As a result, first-phase insulin secretion and total secretion remain comparable to $\text{GJ}+/{\text{K}}_{ATP}+$ conditions, but the frequency of oscillatory secretion is markedly increased. Together, our metamodel reveals the interplay between two complementary regulatory mechanisms of islet insulin secretion: GJ-coordinated islet synchronization and $K^{+}$ channel–mediated single-cell dynamics, each shaping distinct aspects of islet function across scales.

### Identifying and characterizing hub cells as mechanistic drivers of Islet activity and synchronization

**Fig 5.**
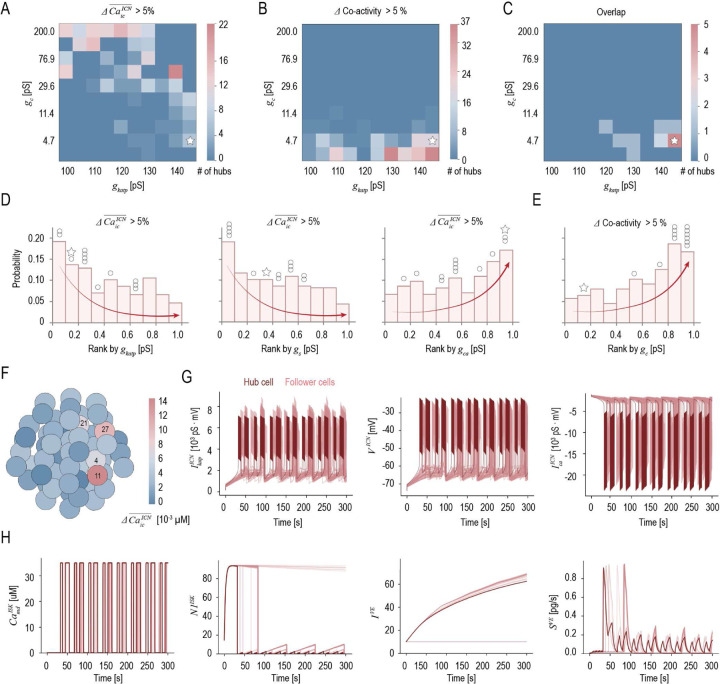
Identification of hub cells using the metamodel (A) Potential hub cells identified based on changes in intracellular $Ca^{2+}$ levels $\left(\Delta \bar{Ca_{ic}^{\text{ICN}}}>5\%\right)$, (B) Loss of islet co-activity (Δ Co-activity > 5%), or (C) both criteria following 300 seconds of glucose stimulation and cell-specific silencing under varying intercellular communication conditions. (D) Rank of ion channel conductance in hub cells governing islet activity relative to all other islet cells, with a score of 1 indicating the highest conductance and 0 the lowest. Shown are ATP/ADP-dependent potassium channel conductance, $g_{\text{kat}}$, slow inhibitory potassium channel conductance, $g_{s}$, voltage-gated ${Ca}^{2+}$ channel conductance, and $g_{ca}$. (E) Rank of gap junction conductance, $g_{c}$, in hub cells governing islet synchronization relative to all other islet cells. (F) Changes in intracellular $Ca^{2+}$ levels upon silencing each β-cells in the islet network under the same conditions as in Fig. 2 (white star in A-D). (G) Ion channel currents and membrane potentials of each β-cell in the islet, with the dark red curve corresponding to the hub cell 11. Shown are ATP/ADP ratio dependent potassium current, $I_{\text{katp}}^{ICN}$, membrane potential, $V^{ICN}$, and voltage-dependent calcium current, $I_{ca}^{ICN}$. (H) ${Ca}^{2+}$ levels within microdomains near the ${Ca}^{2+}$ channels, $Ca_{md}^{ISK}$, amounts of proinsulin aggregates, $N1^{ISK}$, pool of proinsulin aggregates, $I^{V\;E}$, and insulin secretion, $S^{V\;E}$ in individual cells, with the dark red curve corresponding to hub cell 11.

A subset of β-cells (1–10%) has been proposed to act as hubs first responding to glucose stimulation and driving normal islet function [30, 31, 32]. Despite their significance, the defining properties and underlying mechanisms of these hub cells driving islet function remain largely unknown. Using the cell metamodel, we identify and characterize these hub cells, revealing their regulatory mechanisms across scales. We first silence individual β-cells and assess the resulting changes in islet activity and synchronization across varying GJ conductances $\left(g_{c}=4-200\;\text{pS}\right)$ and ${\text{K}}_{ATP}$ channel conductances $\left(g_{\text{katp}}=100-145\;\text{pS}\right)$. Silencing is implemented by hyperpolarizing the target cell *via* binarized passive chloride pump currents, which effectively prevent membrane depolarization [12]. We quantify the number of cells whose silencing results in (i) a reduction in average intracellular $Ca^{2+}$ levels $\left(\Delta {\bar{Ca_{ic}}}^{ICN}\geq \%5\right)$, indicating decreased islet activity; (ii) a reduction in average cell co-activity (Δ Co-activity ≥ %5), reflecting diminished islet synchronization; and (iii) both effects, representing the overlapping subset. Cells whose silencing reduces islet activity emerge along a diagonal in the $g_{c}-g_{\text{katp}}$ space: from few to many as $g_{c}$ increases (stronger coupling) and $g_{\text{katp}}$ decreases (higher excitability)(Fig. 5A). In contrast, cells whose silencing disrupts islet synchronization are primarily observed with small $g_{c}$ (weaker coupling) (Fig. 5B). As expected, in a highly coupled islet, silencing a single β-cell exhibits minimal impact on global synchronization. Notably, only a small subset of cells—13 hubs—affects both when silenced, out of 268 and 256 hubs governing activity and synchronization, respectively (Fig. 5C), suggesting that islet activity and synchronization are largely regulated by distinct subpopulations of hub cells.

To uncover the physiological characteristics of the distinct hub cell types, we compare their ion channel profiles to those of other islet cells (Fig. 5DE, SI Fig. S6AB). Rather than exhibiting excessively high conductance in any single ion channel, hub cells show moderate adjustments across multiple ion channels (Fig. 5D). Specifically, hubs governing islet activity demonstrate (i) relatively lower ATP/ADP-dependent potassium channel conductance, $g_{\text{katp}}$, and slow inhibitory potassium channel conductance, $g_{s}$, which lowers the depolarization threshold by requiring less ATP to close these channels and thus facilitate rapid membrane depolarization upon glucose stimulation; (ii) relatively higher voltage-gated $Ca^{2+}$ channel conductance, $g_{ca}$, which enables robust $Ca^{2+}$ influx upon membrane depolarization. Hubs governing islet synchronization display relatively higher gap junction conductance (Fig. 5E). To further investigate, we examine Metamodel-57 in Fig. 2 (white star in Fig. 5A–C), in which four hub cells are identified and distributed randomly across the islet network, consistent with multiple hub regions observed in islets using fluorescence lifetime imaging [33, 34]. Among them, cell 11 is the most influential as its silencing leads to the largest reduction in islet activity (average intracellular ${Ca}^{2+}$ levels dropping from 0.08μM to 0.07μM) and islet synchronization (average cell co-activity decreasing from 0.74 to 0.67) (white star in Fig. 5D–E). Its low $K^{+}$ channel conductance triggers the earliest ATP/ADP ratio dependent $K^{+}$ current, $I_{\text{katp}}^{\text{ICN}}$, along with other channel currents, producing the earliest and highest membrane potential, $V^{ICN}$, reflecting a rapid response to glucose stimulation (Fig. 5G, SI Fig. S6C). Concurrently, its enhanced ${Ca}^{2+}$ channel conductance promotes robust ${Ca}^{2+}$ influx, resulting in the earliest and highest voltage-dependent calcium current, $I_{ca}$, reflecting strong $Ca^{2+}$ signaling. The rapid $Ca^{2+}$ influx the hub cell induces localized $Ca^{2+}$ increases within microdomains near the $Ca^{2+}$ channels, $Ca_{md}^{ISK}$ (Fig. 5H), triggering rapid insulin vesicle priming and release, as reflected by a rapid increase followed by a decrease in the number of primed vesicles within the microdomain, $N1^{ISK}$, as well as the initial peak in insulin secretion, $S^{V\;E}$. Thus, hub cells not only function as pacemakers driving the activity of follower cells but also could contribute to initiating first-phase insulin secretion. However, proinsulin aggregate synthesis in the hub cell, $I^{V\;E}$ is slower than in follower cells, resulting in minimal $N1^{ISK}$ dynamics and a limited contribution to sustained second-phase insulin secretion, $S^{V\;E}$ (Fig. 5H).

**Fig 6.**
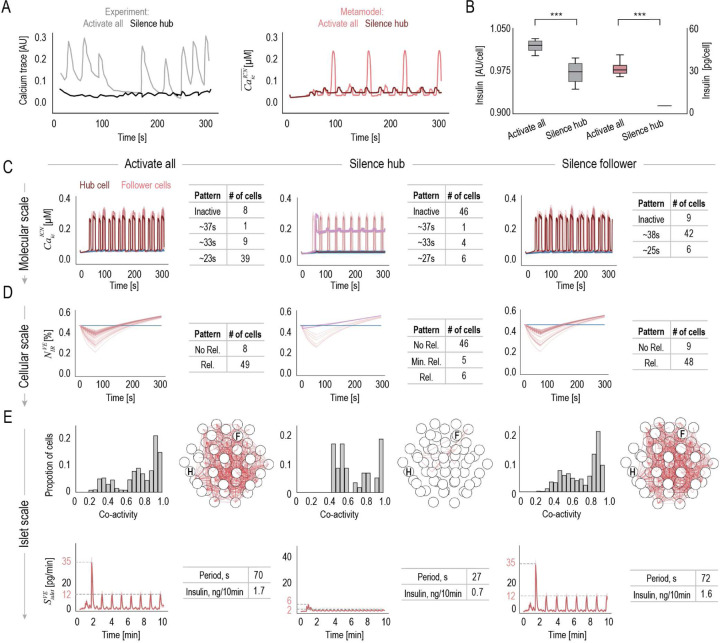
Hub cell function across molecular, cellular and islet scales (A) ${Ca}^{2+}$ traces under activating all and silencing hub cell conditions in optogenetic silencing experiments (gray) [30], compared with the metamodel predictions (red). (B) Insulin secretion under activating all and silencing hub cell conditions, measured experimentally using a fluorescent $Zn^{2+}$ probe (gray) [30], and predicted by the metamodel (red). Metamodel predictions across molecular, cellular, and islet scales under conditions of activating all cells, silencing the hub cell, and silencing follower cells, including (C) intracellular $Ca^{2+}$ level of individual cells, $Ca_{ic}^{ICN}$, the six cells shown in purple exhibit a pattern with a 27s period.(D) The fraction of immediately releasable insulin vesicles, $N_{IR}^{V\;E}$, in individual cells, the same six cells with a 27s pattern are highlighted in purple. (E) Proportion of cells exhibiting varying levels of co-activity with others, identification of cell pairs with co-activity >0.85 within the islet, and the insulin secretion rate of a single islet, $S_{\text{islet}}^{V\;E}$. Shaded regions represent standard deviations (SDs).

We then perform metamodel inference under three conditions: (i) activate all cells, (ii) silence hub cell 11, and (iii) silence follower cell 35. Compared to other cells, silencing the hub cell 11 disrupts oscillatory ${Ca}^{2+}$ patterns (Fig. 6A, SI Fig. S7) and significantly reduces islet insulin secretion (Fig. 6B), as quantified by the cumulative sum of insulin secretion rates, $S^{V\;E}$, from all β-cells, in alignment with experimental observations [30]. Under healthy conditions, at the molecular scale, most cells exhibit oscillatory ${Ca}^{2+}$ patterns with three characteristic periodicities (Fig. 6C). Hub cell 11 is the first to respond to glucose (dark red curve), as previously observed using fluorescence microscopy experiments [35]. At the cellular scale, active cells initially show a decrease in the IRP, followed by gradual replenishment (light pink), while inactive cells plateau (blue) (Fig. 6D). At the islet scale, β-cells exhibit medium-to-high co-activity within the islet network (Fig. 6E). As expected, the hub cell shows zero co-activity with followers, as it activates first, while follower cells experience a delayed activation. The islet insulin secretion, $S_{\text{islet}}$ exhibits a biphasic, oscillatory pattern. Silencing the hub cell leads to a significant reduction in the number of active cells. Notably, 5 out of the 11 remaining active cells maintain relatively high ${Ca}^{2+}$ levels (violet curves in Fig. 6C), indicating sustained activity, yet minimal ${Ca}^{2+}$ oscillation and IRP release (violet curves in Fig. 6D). As a result, silencing the hub cell leads to a reduction in cell co-activity, and two major changes in islet function: a significant reduction in the first phase and nearly complete loss of oscillations, both hallmark phenotypes observed in T2D patients [20]. In contrast, silencing the follower cell has negligible effects compared to activating all cells.

### Potential therapeutic targets for restoring islet function in diabetic phenotypes

**Fig 7.**
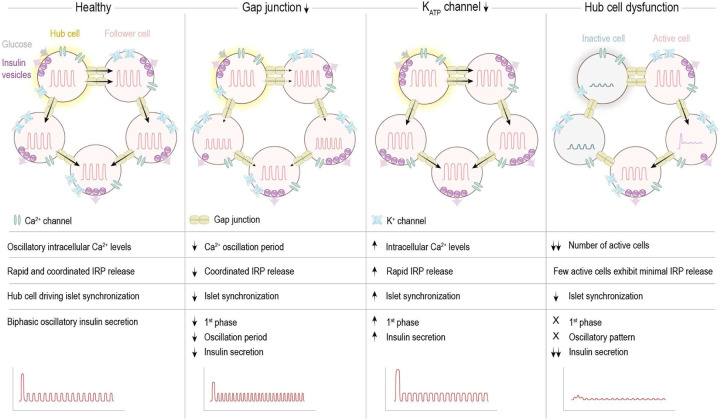
Schematic representation of the role of intercellular communication and hub cells in β-cell dynamics, activity and islet function Illustration of intracellular $Ca^{2+}$ oscillations, the release of insulin vesicles from the immediately releasable pool (IRP), and the biphasic oscillatory insulin secretion of the entire islet under healthy, lowered gap junction conductance, lowered ${\text{K}}_{ATP}$ channel conductance, and hub cell dysfunction conditions.

Our cell metamodel uncovers regulatory mechanisms of β-cell dynamics and islet function (Fig. 7). In a healthy islet, the interplay between gap junction–coupling and $K^{+}$ channel-mediated signaling propagates across scales to regulate islet function. Two types of hub cells deterministically drive islet activity and synchronization. Insulin vesicles in the IRP exhibit rapid and coordinated release, resulting in a biphasic, oscillatory pattern of islet insulin secretion. Reduced GJ conductance leads to shorter and irregular ${Ca}^{2+}$ oscillations, disrupted IRP vesicle depletion, and reduced islet synchronization, resulting in a reduced first phase and an impaired oscillatory pattern in insulin secretion, with lower amplitudes, shorter periods, and an overall reduced secretion amount. In contrast, lowered $K^{+}$ channel dysfunction elevates intracellular $Ca^{2+}$ levels by accelerating the onset and prolonging the duration of each depolarized phase, promoting ultra-rapid the IRP vesicle depletion, enhanced islet synchronization and increased insulin secretion. Moreover, hub cell dysfunction significantly disrupts β-cell activity, abolishes their synchronization, and significantly reduces both the first-phase and oscillatory components of insulin secretion.

One major diabetic phenotype, the reduction in first-phase insulin secretion, may be influenced by two mechanisms: the collective dynamics of $K^{+}$ channels across β-cells, where reduced conductance enhances the initial response, and hub cells that drive islet activity. Another hallmark diabetic phenotype, disrupted oscillatory insulin secretion, may be modulated by the interplay between gap junction–coordinated islet synchronization and $K^{+}$ channel–mediated single-cell dynamics, as well as by hub cells driving islet synchronization. As both gap junctions and $K^{+}$ channels are critical regulators of insulin secretion and glucose homeostasis [36, 22, 37], simultaneously modulating these mechanisms, rather than overactivating or inhibiting individual pathways, could offer a more balanced and effective therapeutic strategy to restore impaired insulin secretion in diabetes. Interestingly, hub cells appear particularly vulnerable to pro-inflammatory and glucolipotoxic conditions, as those found in T2D [30]. Yet, other β-cells may retain the glucose-sensing capacity to emerge as new hubs over time [25]. Thus, promoting the emergence of hub cells may represent a potential strategy to restore islet function in diabetes.

## Discussion

We develop a graph-based metamodeling framework for modeling of complex biological systems and apply it to integrate diverse models of pancreatic β-cell physiology into a cell metamodel, capturing glucose-stimulated insulin secretion at the islet level while resolving molecular and cellular dynamics within individual β-cells. The metamodel accurately reproduces a wide range of experimental data, including fluorescence imaging of cellular structures, and offer mechanistic insights that are unattainable with individual models alone.

Biphasic, oscillatory insulin secretion of pancreatic islets is regulated by the interplay of two mechanisms: gap junctions coordinate β-cell synchronization across the islet network *via* intercellular ion exchange, while $K^{+}$ channels shape the oscillatory dynamics of individual β-cells by modulating the onset and duration of intracellular ${Ca}^{2+}$ bursts. Two types of hub cells further regulate islet function: one driving islet activity, distinguished by reduced $K^{+}$ and elevated ${Ca}^{2+}$ channel conductance, and another promoting islet synchronization, characterized by increased gap junction conductance relative to other cells. Perturbing channel conductance and hub cell activity recapitulates diabetic phenotypes and highlight potential therapeutic targets. Notably, previous studies have reported that hub cells may exhibit high levels of glucokinase (GK) expression, making them more sensitive to increases in glucose concentration [30]. Although the current cell metamodel does not explicitly include GK dynamics, its prediction of relatively low $K_{\text{ATP}}$ channel conductance is consistent with the effect of elevated GK activity, as increased ATP levels trigger the closure of $K_{\text{ATP}}$ channels. Next, we plan to integrate additional models capturing other β-cell components, as well as interactions among α-,β-, and γ-cells within an islet [38], to further enhance the accuracy, completeness, and predictive power of the metamodel.

The graph-based metamodeling framework developed in this study overcomes critical challenges in cell modeling by accurately unifying representations of diverse inputs with robust uncertainty quantification, effectively integrating surrogates across timescales through a standardized coupling strategy, and facilitating efficient inference for complex model systems. Although promising, further developments are required to achieve full automation. First, we should accommodate time-variant (*eg*, t+2,t+3), higher-order process functions to better capture complex statistical dependencies. To address this, techniques such as high-order Recurrent Neural Networks (RNNs) for long-term dependencies [39] and Markov decision processes for sequential decision-making in stochastic environments [40] could be incorporated. Second, we could benefit from clustering algorithms to automate the construction of the coupling PGM graph. Machine learning approaches, such as basisVAE, utilize basis functions to cluster similar features, facilitating the identification of connecting variables and their conditional dependencies [41]. Additionally, exploring a broader range of connecting variables and alternative graph topologies—such as serial, convergent, or hybrid graphs—could potentially improve the coupling accuracy. Third, we should more effectively integrate models with large variations in their time scales. This could be addressed by employing Bayesian hierarchical models [42] or hierarchical physical-informed neural networks [43], allowing the system to learn cross-timescale patterns while maintaining the established hierarchies. Finally, improving computational efficiency is crucial for modeling complex systems. Non-parametric methods or variational inference approaches could significantly improve efficiency, particularly for high-dimensional and computationally intensive models [44].

In summary, the cell metamodel bridges molecular and cellular dynamics to emergent islet function, uncovering mechanistic drivers of diabetics and highlighting potential therapeutic targets. More broadly, the graph-based metamodeling framework provides a generalizable approach for integrating models across diverse representations and spatiotemporal scales, enabling the modeling of complex biological systems and advancing our understanding of cell biology and disease.

## Resource Availability

### Lead contact

Further information and requests for resources should be directed to and will be fulfilled by the lead contact, Liping Sun (sunlp@shanghaitech.edu.cn).

### Data and code availability

The software, input files, and example output files for the present work are available at https://github.com/SunLab-SH/GraphMM. All SIM cell images are available from the corresponding author upon request.

## Methods

### Standardizing physiological conditions across models

We construct a multiscale β-cell metamodel by integrating 57 ODE-based Vesicle Exocytosis (VE) models, 57 compartment-based Insulin Secretion Kinetic (ISK) models, and 1 network-based Islet Cell Network (ICN) model comprising 57 β-cells (SI Text 1.1–1.3, SI Table S1–3). For each of the 57 cells in the ICN model, the membrane potential values, $V^{ICN}$, are used to determine the periodic cycle duration, $t_{\text{cycle}}$, and the rest phase duration, $t_{off}$, for the VE and ISK models. For the VE model, the insulin vesicle fusion rate coefficient at timestep $t,\rho_{t}$ is defined as:

(1) $$\rho_{t}=\left\{\begin{array}{ll} \rho_{\text{basal}}, & \text{for}\;t\notin \left[n\cdot t_{\text{cycle}},n\cdot t_{\text{cycle}}+t_{\text{off}}\right]\\ \rho_{\text{basal}}+\rho_{\text{hat}}\left(1-e^{-\epsilon \left(t-t_{1}\right)}\right)+S_{p}\left(t-t_{1}\right), & \text{for}\;t\in \left[n\cdot t_{\text{cycle}},n\cdot t_{\text{cycle}}+t_{\text{off}}\right] \end{array}\right.$$

where $\rho_{\text{basal}}$ and $\rho_{\text{hat}}$ are the basal and maximal fusion rate; n is the number of periodic cycles, each comprising a burst phase followed by a rest phase; t and $t_{1}$ are the current timestep and the onset of the burst phase; ϵ is the rate of rise in membrane potential, and $S_{p}$ is the slope of the incremental fusion rate over time. For the ISK model, the membrane potential at timestep $t,V_{t}^{ISK}$ is defined as:

(2) $$V_{t}^{ISK}=V_{\text{rest}}+\left(V_{\text{burst}}-V_{\text{rest}}\right)\left[H\left(mod\left(t,t_{\text{cycle}}\right)-\epsilon \right)-H\left(mod\left(t,t_{\text{cycle}}\right)-t_{\text{off}}\right)\right]$$

where $V_{\text{rest}}$ and $V_{\text{burst}}$ are rest and burst membrane potentials, respectively; H is the Heaviside step function, which switches between phases based on the modulo operation of the timestep t relative to $t_{\text{cycle}}$, offset by $t_{off}$; and $\epsilon \left(\sim 10^{-10}\right)$ is a small offset introduced to ensure numerical precision.

### Graph-based metamodeling framework

We adopt Boltzmann’s concept of phase spaces, which encompasses all possible values of position and momentum parameters. Any biological system, regardless of complexity, can be represented within a high-dimensional ‘reference phase space’ that encodes complete information about all constituent atoms and their immediate environment in four dimensions (*ie*, three spatial coordinates and time) over the system’s lifetime. However, directly modeling such systems within the reference phase space is computationally infeasible. For instance, simulating the trajectory of a typical eukaryotic cell, containing 10^14^ atoms over 24 hours using all-atom molecular dynamics at a 2-femtosecond timestep, would require at least 10^33^ dimensions. In practice, tractable models are constructed in ‘derived phase spaces’—lower-dimensional representation of certain aspects of the modeled system. Metamodeling integrates models from multiple derived phase spaces into a unified meta phase space, thereby enabling a tractable approximation of the reference phase space. It proceeds through three stages by (i) converting them into universal probabilistic representations, (ii) coupling them across time scales using a standardized strategy, and (iii) harmonizing them through approximate model inference, as detailed below (SI Fig. S1).

#### Stage1: Converting diverse input models into universal surrogates

An input model can be seen as a joint probability distribution over its variables. Similarly, data can also be interpreted as a data model, where each variable is associated with a marginal probability distribution. For input models that lack explicit uncertainty (*eg*, ODE models), their distributions are estimated using the experimental data employed during construction (SI Text 2). A surrogate model is a probabilistic version of the input model, represented as a Probabilistic Graphical Model (PGM), specifically a State-Space Model (SSM), to capture complex system dynamics through state transitions [45].

We first extract the state variables $\left(Z^{1}:a^{1},b^{1}\right)$ and their corresponding observation variables $\left(O^{1}:a^{O1},b^{O1}\right)$ from the input model, $M^{1}$. We then define a process model, $p\left(Z_{t+1}^{1}|Z_{t}^{1}\right)$, to describe intra- and inter-timestep statistical dependencies among state variables:

(3) $$p\left(Z_{t+1}^{1}|Z_{t}^{1}\right)=P\left(a_{t+1}^{1},b_{t+1}^{1}|a_{t}^{1},b_{t}^{1}\right)=N\left(f\left(a_{t}^{1},b_{t}^{1}\right),\phi_{t}^{1}\right)$$

where $f\left(a_{t}^{1},b_{t}^{1}\right)$ is the forward function derived from the input model (*eg*, differential equations) or inferred using regression fitting or machine learning approaches [46]. The process model is corrupted by transition noise, $\phi_{t}^{1}$, to account for stochasticity. Next, we define an observation model, $p\left(O_{t}^{1}|Z_{t}^{1}\right)$, to represent noisy observations of the state variables. This model uses experimental data when available or an identical function to align predicted and observed states:

(4) $$p\left(O_{t}^{1}|Z_{t}^{1}\right)=P\left(a_{t}^{O1},b_{t}^{O1}|a_{t}^{1},b_{t}^{1}\right)=N\left(g\left(a_{t}^{1},b_{t}^{1}\right),\epsilon_{t}^{1}\right)$$

where $g\left(a_{t}^{1},b_{t}^{1}\right)$ is identical to the forward function to $f\left(a_{t}^{1},b_{t}^{1}\right)$, which relates predicted and observed states. The observation model is corrupted by emission noise, $\epsilon_{t}^{1}$, to capture observation uncertainty. Transition and emission noises are time-variant, ensuring that standard deviation (SD)-to-mean ratios of the surrogate model variables closely align with those observed in the experimental data.

#### Stage2: Standardized coupling across time scales

We develop a standardized strategy for coupling surrogate models across time scales using nonlinear and linear mappings to unify surrogates onto a universal timestep, rather than relying solely on common timesteps. The universal timestep enables consistent inference across models with different temporal resolutions. It is chosen between the minimum timestep and the least common multiple of the input model timesteps, ensuring accurate forward computation with the ODE solver [47]. For surrogate models with small timesteps, the process model is redefined at the universal timestep, while the observation model is redefined by directly assigning variable distributions at universal timesteps from the original observations at smaller timesteps, facilitating nonlinear mapping of both models. For surrogate models with large timesteps, the process model is redefined at the universal timestep to achieve a nonlinear mapping. The observation model is redefined through linear interpolation if experimental data are available; otherwise, a nonlinear mapping is applied based on the identical function.

We construct the coupling PGM graph over the state variables of the process models at each universal timestep using a standardized strategy. First, connecting variables from different surrogate models are identified based on identical semantics or the highest Pearson correlation coefficient among all pairs of surrogate model variables, *e g*, $b^{1}$ and $b^{2}$ from models $M^{1}$ and $M^{2}$. These variables are assumed to follow Gaussian distributions, $p\left(b_{t}^{1}\right)=N\left(\mu^{b1},\sigma^{b1}\right)$ and $p\left(b_{t}^{2}\right)=N\left(\mu^{b2},\sigma^{b2}\right)$. Second, an explicit coupling variable $c_{t}^{12}$ is introduced, with its prior distribution, $p\left(c_{t}^{12}\right)$, defined as a mixture function of the connecting variable distributions [48], ensuring that $p\left(c_{t}^{12}\right)$ remains a unimodal Gaussian distribution. Unless relative model confidence has been rigorously assessed, equal weights are assigned to the connecting variable distributions in the mixture function, as follows:

(5) $$p\left(c_{t}^{12}\right)=N\left(h\left(b_{t}^{1},b_{t}^{2}\right),\rho_{t}\right),\;h\left(b_{t}^{1},b_{t}^{2}\right)=0.5\mu^{b1}+0.5\mu^{b2},\;{\left(\rho_{t}\right)}^{2}=0.5{\left(\sigma^{b1}\right)}^{2}+0.5{\left(\sigma^{b2}\right)}^{2}+0.5\times 0.5{\left(\mu^{b1}-\mu^{b2}\right)}^{2}$$

Third, coupling is implemented by constructing ‘head-to-tail’ graph structures with direct edges from the coupling variable to the connecting variables, *eg*, $b_{t}^{1}\leftarrow c_{t}^{12}\to b_{t}^{2}$. In probabilistic graphical model terms, variables in the reference phase space (*ie*, reference variables) serve as parent nodes and those in derived phase spaces (*ie*, derived variables) as child nodes. Parent-to-child edges establish statistical dependencies, with reference variables functioning as latent truths that contextualize the derived variables across input models.

The corresponding conditional probability distributions (*eg*, $p\left(b^{1}|c^{12}\right)$ and $p\left(b^{2}|c^{12}\right)$) are parameterized according to the relative overlaps between the distributions of the coupling variable and the connected variables, defined as weighted sums of their distributions from the previous timestep:

(6) $$p\left(b_{t+1}^{1}|c_{t}^{12},a_{t}^{1},b_{t}^{1}\right)=N\left(\omega_{t}^{1}y_{t}+\left(1-\omega_{t}^{1}\right)f^{1}\left(a_{t}^{1},b_{t}^{1}\right),\phi_{t}^{1}\right),\;p\left(b_{t+1}^{2}|c_{t}^{12},a_{t}^{2},b_{t}^{2}\right)=N\left(\omega_{t}^{2}y_{t}+\left(1-\omega_{t}^{2}\right)f^{2}\left(a_{t}^{2},b_{t}^{2}\right),\phi_{t}^{2}\right)$$

where $f^{1}$ and $f^{2}$ are the forward functions in the process moodel, $\phi_{t}^{1}$ and $\phi_{t}^{2}$ are the transition noises, and $\omega_{t}^{1}$ and $\omega_{t}^{2}$ are weights determined by the relative overlaps between the distributions of the coupling variable and the connecting variables. Thus, we obtain a meta PGM graph that includes all surrogate model variables, Z, and coupling variables, C. The resulting metamodel distribution at time t, given their observations is expressed as:

$$p\left(Z_{t}|O_{1:t}^{1},O_{1:t}^{2}\right)\propto p\left(Z_{t},O_{t}^{1},O_{t}^{2}|O_{1:t-1}^{1},O_{1:t-1}^{2}\right)\;=\int_{Z_{t-1}}\left[\int_{C_{t}}p\left(Z_{t},C_{t}|Z_{t-1}\right)dC_{t}\right]p\left(O_{t}^{1},O_{t}^{2}|Z_{t}\right)p\left(Z_{t-1}|O_{1:t-1}^{1},O_{1:t-1}^{2}\right)dZ_{t-1}$$

Here, the conditional probability, $p\left(Z_{t},C_{t}|Z_{t-1}\right)$, determines how the coupling variables update the surrogate state variables, and how surrogate state variables update each other.

#### Stage3: Efficient metamodel inference

We perform approximate inference over the metamodel using factor graphs, enabling efficient message passing through the the complex model structure, *eg*, high-order cliques (multiple connecting variables linked to the same coupling variable) and loopy graphs (multiple coupling variables linked to the same connecting variables). At each timestep t, we introduce factors, $f_{i}\left(Z_{t}^{i},c_{t}^{i}\right)$, to capture subsets of distributions and statistical dependencies between surrogate state variables $Z_{t}^{i}$ and coupling variables $C_{t}^{i}$. The metamodel distribution can be obtained as the product of all the factors:

$$\frac{1}{K}\prod_{i}f_{i}\left(Z_{t}^{i},C_{t}^{i}\right)$$

where K is a normalization constant. The *predict–update* cycle is then recursively applied within and across timesteps. In the *predict* step, the prior distribution of the state variables at timestep t given all observations up to the previous timestep, $O_{1:t-1}$, is computed as:

(7) $$P\left(Z_{t}|O_{1:t-1}\right)=\int_{Z_{t-1}}\int_{C_{t}}P\left(Z_{t},C_{t}|Z_{t-1}\right)dC_{t}P\left(Z_{t-1}|O_{1:t-1}\right)dZ_{t-1}$$

In the *update* step, the posterior distribution of the state variables at timestep t, given the observations at the same timestep, $O_{t}$, is computed using Bayes’ theorem as:

(8) $$P\left(Z_{t}|O_{1:t}\right)=\frac{P\left(O_{t}|Z_{t}\right)P\left(Z_{t}|O_{1:t-1}\right)}{P\left(O_{t}|O_{1:t-1}\right)}$$

The likelihood $P\left(O_{t}|Z_{t}\right)$ represents the conditional probability of an observation given the predicted state at timestep t, while the normalizing constant $P\left(O_{t}|O_{1:t-1}\right)$ denotes the probability of the observation. The distributions of metamodel variables are then estimated using Unscented Kalman Filters (UKFs) for Gaussian distributions and Particle Filters (PF) for non-Gaussian distributions [49]. Note that PFs provide higher accuracy but are less efficient and susceptible to particle collapse [50]. Our graph-based metamodeling framework is publicly available (https://github.com/SunLab-SH/GraphMM), along with a tutorial based on a synthetic metamodel (SI Text 3, SI Fig. S3), to support collaborations across disciplines.

### Structured Illumination Microscopy (SIM) collection and analysis

Fixed INS-1E β-cell samples were prepared by culturing the cells on glass coverslips (ibidi) followed by transfection with plasmids to label ISGs and actin filaments. The cells were subsequently stimulated with glucose to establish three experimental conditions: 2.8 mM glucose for 30 minutes, 16.7 mM glucose for 5 minutes, and 16.7 mM glucose for 30 minutes, as previously described. The samples were then washed three times with 1× PBS at room temperature and fixed with 4% paraformaldehyde for 20 minutes. Following fixation, the cells were washed three additional times with 1× PBS and incubated in 2 mL of Hoechst 33342 staining solution (1 g/mL, Cell Signaling) for 5 minutes. After staining, samples were washed three times with 1× PBS, and a glass coverslip was mounted with a drop of ProLong^™^ Glass Antifade Mountant (Thermo Fisher Scientific) before being placed onto a glass slide. The samples were maintained at room temperature for 48 hours and subsequently stored at 20°C in the dark. Super-resolution fluorescence images were acquired using structured illumination microscopy (SIM) mode on a Zeiss Elyra 7 with Lattice SIM, equipped with a PCO edge 4.2 sCMOS camera and a Plan-Apochromat 63x/1.4 Oil DIC M27 objective. Image acquisition was managed using Zen 64-bit software (version 3.0 SR FP1 black, Carl Zeiss). The fluorescence channels utilized were Hoechst 33342 (Ex 345–355 nm, Em 450–460 nm), EGFP (Ex 483–493 nm, Em 502–512 nm), and mCherry (Ex 582–592 nm, Em 605–615 nm). The resolution of the SIM data was determined to be 31.3 nm in the x and y dimensions, and 90.9 nm in the z dimension. Following instance segmentation of insulin vesicles using ImageJ (version 1.53), docked vesicles were defined as those whose centers lie within one vesicle radius of the plasma membrane (PM), with the radius estimated based on each vesicle’s volume.

## Supplementary Material

Supplementary Files

This is a list of supplementary files associated with this preprint. Click to download.

- SI.pdf
